# Clustering and correlates of screen-time and eating behaviours among young adolescents

**DOI:** 10.1186/s12889-017-4441-2

**Published:** 2017-05-31

**Authors:** Natalie Pearson, Paula Griffiths, Stuart JH Biddle, Julie P Johnston, Sonia McGeorge, Emma Haycraft

**Affiliations:** 1 School of Sport, Exercise & Health Sciences, National Centre for Sport & Exercise Medicine, Loughborough University, Loughborough, Leicestershire, LE11 3TU UK; 2 Institute for Resilient Regions, University of Southern Queensland, Queensland, Australia; 3 Department of Sport Science, School of Science and Technology, Nottingham Trent University, Nottingham, UK

**Keywords:** Screen-time, Fruit, Vegetables, Energy-dense snacks, Clustering, Correlates, Adolescents

## Abstract

**Background:**

Screen-time and eating behaviours are associated in adolescents, but few studies have examined the clustering of these health behaviours in this age group. The identification of clustered health behaviours, and influences on adolescents’ clustered health behaviours, at the time when they are most likely to become habitual, is important for intervention design. The purpose of this study was to assess the prevalence and clustering of health behaviours in adolescents, and examine the sociodemographic, individual, behavioural, and home social and physical environmental correlates of clustered health behaviours.

**Methods:**

Adolescents aged 11–12 years (*n* = 527, 48% boys) completed a questionnaire during class-time which assessed screen-time (ST), fruit and vegetable (FV), and energy-dense (ED) snack consumption using a Food Frequency Questionnaire. Health behaviours were categorised into high and low frequencies based on recommendations for FV and ST and median splits for ED snacks. Adolescents reported on their habits, self-efficacy, eating at the television (TV), eating and watching TV together with parents, restrictive parenting practices, and the availability and accessibility of foods within the home. Behavioural clustering was assessed using an observed over expected ratio (O/E). Correlates of clustered behaviours were examined using multivariate multinomial logistic regression.

**Results:**

Approximately 70% reported having two or three health risk behaviours. Overall, O/E ratios were close to 1, which indicates clustering. The three risk behaviour combination of low FV, high ED, and high ST occurred more frequently than expected (O/E ratio = 1.06 95% CI 1.01, 1.15. Individual, behavioural, and social and physical home environmental correlates were differentially associated with behavioural clusters. Correlates consistently associated with clusters included eating ED snacks while watching TV, eating at the TV with parents, and the availability and accessibility of ED snack foods within the home.

**Conclusions:**

There is a high prevalence of screen time and unhealthy eating, and screen time is coupled with unhealthy dietary behaviours. Strategies and policies are required that simultaneously address reductions in screen time and changes to habitual dietary patterns, such as TV snacking and snack availability and accessibility. These may require a combination of individual, social and environmental changes alongside conscious and more automatic (nudging) strategies.

**Electronic supplementary material:**

The online version of this article (doi:10.1186/s12889-017-4441-2) contains supplementary material, which is available to authorized users.

## Background

The topic of adolescent health has been considered as paradoxical because, while adolescence is often a healthy stage of life, many young people form unhealthy behavioural habits. Health behaviours such as low intakes of fruit and vegetables, high intakes of energy-dense snack foods, and excessive sedentary behaviour are commonly established during this life stage [1–3], and these behaviours have been shown to persist into adulthood [3, 4]. Modifiable health behaviours such as those just mentioned, have been associated with overweight and obesity, cardio-metabolic risk, and poorer mental and physical health [5–8]. Furthermore, such health behaviours do not occur in isolation, and there is a growing body of research suggesting that ‘unhealthy’ behaviours such as low fruit and vegetable consumption and sedentary screen-time co-occur as risk behavioural clusters in adolescents [9, 10]. The odds of having multiple risk behaviours increase over the course of development, especially during the teenage years [11]. While individual adolescent health behaviours are of public health concern as independent behaviours, arguably it may be more important to look at the extent of ‘clustering’ of unhealthy behaviours (i.e. screen time and low fruit and vegetable consumption). As young people form clusters of unhealthy behaviours that contribute to the development of disease biomarkers, they launch synergistic trajectories towards chronic disease [12, 13]. This parody makes adolescents’ lifestyle choices, particularly those relating to health behaviours, an important area for public health consideration.

Identifying the interrelationship (or clustering) of health risk behaviours in adolescents, and focusing on the contextual factors that may either increase risk or operate as protective factors, is imperative for the development of targeted prevention initiatives. However, the predominant focus on individual health behaviours tends to dominate the literature to date. Studies examining the prevalence and influences on individual health behaviours are in abundance, while there are far fewer studies examining the clustering, or the co-occurrence of behaviours [14–18]. Furthermore, we know very little about the contextual factors that influence the clustering of these health behaviours [19]. A recent study by Elsenberg and colleagues [15] found evidence of clustering of screen time and unhealthy dietary behaviours in British children, and that older children and children who attended a school with a physical activity or diet related policy were more likely to have poorer health behaviour risk scores. Whereas girls, participants with siblings, and those with more highly educated parents were less likely to have a poor health behaviour profile [15]. Furthermore, Hardy et al. [17] found evidence of clustering of sedentary behaviour, physical activity and dietary behaviours in Australian adolescents, particularly among adolescents from low income households. To date, research examining factors associated with clustering of health behaviours has focussed on sociodemographic predictors [19].

While sociodemographic factors are important for identifying subgroups for targeted intervention approaches, there are a multitude of other factors that could be of importance in the prevention or promotion of adolescent health behaviours. There is growing support of the use of social–ecological models in understanding health behaviours [20]. Such models posit that factors at the individual (e.g. self-efficacy), social (parental modelling of screen behaviours), and physical (e.g. home availability of foods) environmental levels interact to influence health behaviours [21] such as eating behaviours and sedentary screen-time. However, whether factors at the individual, social and physical levels are associated with clusters of health behaviours is understudied. Therefore, to further understand clustering of health risk behaviours, this paper examines the prevalence and clustering of screen-time and eating behaviours in a sample of young adolescents. Additionally, we adopted a novel approach by investigating the sociodemographic, individual, behavioural, and home social and physical environmental correlates of clustered health behaviours in this group.

## Methods

### Study procedure

Cross-sectional data were collected between May 2013 and June 2014. Study procedures were approved by the Loughborough University Ethical Advisory Committee. Data were obtained from young adolescents in their first year (Year 7) of secondary school (aged 11–12 years) recruited from four secondary schools in the East Midlands region of the UK. All students in Year 7 of participating schools were eligible and received an information leaflet to take home for a parent or guardian with details of the study (*n* = 683). Under existing ethical guidelines, it was necessary to seek written consent from parents for each child’s participation, and no information could be accessed regarding characteristics of non-respondents. In total, 562 parents provided consent (82% response rate). Adolescent participants provided assent before completing written questionnaires during class time, and 527 were present on the data collection days and completed the questionnaire (77% response rate).

## Measures

Participants completed questionnaires during a school lesson under the supervision of trained researchers and class teachers.

### Eating behaviours

Consistent with large scale studies of eating behaviours and dietary intake, food intake was assessed using a Food Frequency Questionnaire which was based on previously validated indices of food intake [22] but options were reduced to focus on the specific foods of interest (namely, fruit, vegetables, and energy-dense snacks) and assessed intake frequency during the past week. Adolescents indicated how frequently they consumed 18 food items during a usual week. Seven response categories ranged from ‘never’ to ‘more than three a day’. The frequency of consumption of the 18 food items in a usual week was converted to a daily equivalent, which is an established method [23] that has been used successfully in other dietary studies [24, 25]. Daily equivalents were calculated as follows: never (0·00 per d); 1–2 days a week (0·2 per d); 3–4 days a week (0·5 per d); 5–6 days a week (0·7 per d); once a day (1.0 per d); twice a day (2.0 per d); three or more a day (3.0 per d). The daily intake of fruit, vegetables, and energy-dense snacks was calculated by summing the daily equivalence for the food items in each food group. The estimated daily intake of ‘fruit’ included the summed equivalence of five fruit items (apples, bananas, oranges, grapes, and other fruit), the daily equivalent of ‘vegetables’ included the summed equivalence of five vegetable items (carrots, peas, broccoli, salad, and other vegetables), the daily equivalence of ‘energy-dense snacks’ included the summed equivalence of eight snack food items (potato crisps, snack crackers, sweets (candy), chocolate, chocolate biscuits, regular biscuits, muffins/cakes, and cereal bars). For the present research question, fruit and vegetables were combined to create a composite ‘fruit and vegetable’ score. Children with a frequency of consumption of fruit and vegetables of five or more were coded as meeting the current fruit and vegetable guidelines of five or more a day [26] (high or low FV). Currently there are no guidelines for the consumption of energy-dense (ED) snack foods, therefore, frequency of consumption of energy-dense snacks were split at the median (a frequency of consumption of 2.8 per day) to create a high and low ED category.

### Screen-time

Adolescents reported the time (in hours and minutes) that they spent watching TV and watching videos/DVDs on a usual school day and on a usual weekend day using an adaptation of the Adolescent Sedentary Activity Questionnaire (ASAQ) [27, 28]. Time spent watching TV and watching videos/DVDs was converted into minutes per school day and weekend day respectively. Weighted mean duration of each behaviour per day ((5*schoolday + 2*weekend day)/7) was derived and summed to provide a measure of screen-time (ST). Adolescents accumulating an average of less than 2 h of TV/DVDs per day were coded as meeting established guidelines regarding screen viewing [29]. The value of 2 h ST per day was used to classify participants into high and low ST groups.

### Demographic, individual, behavioural, social, and physical environmental factors

#### Demographics

Adolescents self-reported their age, sex, ethnicity, whether or not they had siblings living at home, the adults they lived with at home, and their home postcode. Adolescents were coded as ‘male’ or ‘female’, aged ‘11’ or ‘12’ years, ‘White/White British’, ‘Asian/Asian British’ or ‘other’, as having ‘one or more’ or ‘no’ brothers and/or sisters respectively, living at home with their ‘mother and father’ or ‘other’ adults. Socioeconomic position (SEP) was determined using the Index of Multiple Deprivation (IMD), a measure of compound social and material deprivation, calculated from a variety of data including income, employment, health, education, and housing. It is based on the postcode of the participant’s home, and thus represents an area level approximation of SEP. Adolescents were coded as ‘low’, ‘middle’, or ‘high’ SEP based on their IMD.

#### Individual

Adolescents were asked four questions about their habits for eating snack foods in front of the television using the previously validated Self-Report Behavioural Automaticity Index (SRBAI) [30]: ‘eating energy-dense (ED) snack foods (e.g. chocolate/biscuits/crisps) while watching television (TV) is something I do automatically’; ‘… without having to remember’; ‘… without thinking’; ‘… before I realise I’m doing it’. They were asked the same four questions regarding eating fruit and vegetables in front of the TV, and regarding habit for watching TV. Response options were given on a five-point Likert scale, ranging from (1) ‘strongly disagree’ to (5) ‘strongly agree’. Responses were summed separately to provide three habit scores; one for eating ED snacks in front of the TV (Cronbach’s α = 0.86), one for eating fruit and vegetables in front of the TV (Cronbach’s α = 0.91), and one for watching TV (Cronbach’s α = 0.74). Each habit score was dichotomised at the median to create a ‘high’ and ‘low’ habit score. See Additional file 1: Table S1 for all median scores, descriptions and distributions of all predictor variables.

Based on a previously used scale [24], adolescents were asked six questions about their self-efficacy for reducing their energy-dense snack food consumption (i.e. snacks including chocolate, crisps, biscuits, and sweets (candy)): ‘How sure are you that you could not eat snack foods when you’re with your friends’; ‘…you’re with your family’; ‘…after school’; ‘…when you’re alone’; ‘…when you’re bored’; ‘…when you’re feeling down’. They were asked the same six questions about not eating snack foods in front of the TV, about eating more fruit and vegetables, and about reducing their TV viewing. Response options were given on a five-point Likert scale, ranging from (1) ‘not at all sure’ to (5) ‘very sure’. Responses were summed separately to provide four self-efficacy scores; one for not eating energy-dense snacks (Cronbach’s α = 0.89), one for not eating energy-dense snacks in front of the TV (Cronbach’s α = 0.88), one for eating more fruit and vegetables (Cronbach’s α = 0.90), and one for reducing TV viewing (Cronbach’s α = 0.73). Each self-efficacy score was dichotomised at the median to create a ‘high’ and ‘low’ self-efficacy score.

#### Behavioural

Adolescents were asked how often they ate breakfast, lunch, dinner, energy-dense snacks, and fruit and vegetables while also watching the TV during a typical week using an adaptation of a previously used questionnaire by Matheson et al. [31]. Response options were given on a four-point Likert scale ranging from (1) ‘Never’ to (4) ‘every day’. The frequency of consumption of the meals and snacks while watching TV was coded as ‘2 or less days a week’ and ‘3 or more days a week’.

#### Social environmental

Adolescents were asked how often, during a typical week, they did the following activities together with their parents: ate breakfast in front of the TV, dinner in front of the TV, snacks in front of the TV, and watched TV. Response options were given on a five-point Likert scale ranging from (1) ‘Never/less than once a week’ to (5) ‘Every day’. The frequency of consumption of the meals and snacks with parents was coded as ‘less than twice a week’ and ‘2 or more times a week’.

Adolescents were asked seven questions regarding parental food related restriction using items from the Kid’s Child Feeding Questionnaire [32, 33] (e.g. ‘Does your parent ever say things like “you've had enough to eat now, you need to stop”?’). Response options were given on a three-point Likert scale: (1) ‘No’, (2) Sometimes, and (3) ‘Yes’. Scores of the seven items were summed and divided by seven to create the ‘restriction’ score (Cronbach’s α = 0.71), which was dichotomised at the median to create a ‘high’ and ‘low’ score.

Adolescents were asked three questions regarding their perceptions of their parents’ restriction of screen-media in the home. Response options were provided on a 3-point Likert scale: (1) No, (2) Sometimes, and (3) Yes. Items related to restriction of screen-media in the home included, for example, ‘my parents limit how much television I can watch’. The score of the three items were summed and divided by three to create the ‘screen-time restriction’ score (Cronbach’s α = 0.73), which was dichotomised at the median to create a ‘high’ and ‘low’ score.

#### Physical environmental

Adolescents were asked whether or not they had a TV in their bedroom. Response options were ‘yes’ and ‘no’.

Adolescents were asked four questions regarding availability of energy-dense snacks in the home in the past week (e.g. ‘how frequently were the following items available to you at home last week’: cakes/biscuits, crisps, chocolates, sweets), and two questions regarding the availability of fruit and vegetables (fruit and vegetables). Response options were given on a four-point Likert scale ranging from (1) ‘Never/rarely’ to (4) ‘Always’. Scores of the four energy-dense snacks were summed to create the ‘home availability of energy-dense snacks’ score (Cronbach’s α = 0.84) and scores of the fruit and vegetables were summed to create the ‘home availability of fruit and vegetables’ score (Cronbach’s α = 0.83), which were dichotomised at the median to create a ‘high’ and ‘low’ score.

Adolescents were asked two questions regarding the accessibility of energy-dense snacks in the home and four questions regarding accessibility of fruit and vegetables in the home in the past week (e.g. ‘in the past week, were there any fruits that were prepared and ready for you to eat as part of a meal or snack?’). Response options were given on a three-point Likert scale: (1) No, (2) Sometimes, and (3) Yes. Scores of the two energy-dense snacks questions were summed to create the ‘home accessibility of energy-dense snacks’ score (Cronbach’s α = 0.71), and scores of the four fruit and vegetable questions were summed to create the ‘home accessibility of fruit and vegetables’ score (Cronbach’s α = 0.70), which were dichotomised at the median to create a ‘high’ and ‘low’ score.

## Statistical analysis

Analysis was conducted using Stata V12 (Stata, College Station, TX). Sample characteristics were summarised using descriptive statistics.

Prevalence of health risk behaviours were investigated in the study population. Behavioural clustering of two or more health risk behaviours was determined by the ratio of the observed to the expected prevalence of one, two, and three simultaneously occurring risk behaviours, as described previously [15, 34]. Observed prevalence was calculated as the number of participants that did or did not meet guideline levels for each health behaviour divided by the total number of participants (e.g. the proportion of children that had low fruit and vegetable consumption and high TV/DVD time, but consumed less energy-dense snacks than the median). The expected prevalence for single behaviours was calculated as the proportion of participants not meeting a specific guideline multiplied by the proportion of participants that met the guidelines for all remaining behaviours (e.g. the proportion of children that consumed above the median amount of energy dense snacks multiplied by the proportion that had low TV/DVD time, and the proportion that had high levels of fruit and vegetable intake). The expected prevalence for multiple health behaviours was calculated by multiplying the proportion of participants that did not meet guideline levels for a specific set of behaviours and the proportion that met guideline levels for the remaining behaviours. The difference between the observed and the expected prevalence (O/E) was calculated to examine whether health behaviours co-occurred at a higher or lower rate than would be expected if there was no association between behaviours. Ninety five percent confidence intervals were calculated using bootstrap techniques. Observed over expected ratios >1 are indicative of clustering.

Five behavioural cluster categories were created and coded on the basis of met/unmet guidelines: 0: one or no risk behaviours; 1: Low FV / high ED; 2: High ST / Low FV; 3: High ST / High ED; 4: 3 risk behaviours. The proportion of adolescents in each behavioural cluster was compared by gender using Pearson chi-square tests of significance. Multinomial logistic regression analyses were conducted to examine the likelihood of being in each of these categories according to demographic, individual, behavioural, and home social and physical environmental variables. The ‘one or no risk behaviours’ category was used as the referent category. Demographic, individual, behavioural, and home social and physical environmental variables that were significantly associated with combinations of risk behaviours in the univariate multinomial logistic regression analyses were entered into multivariate multinomial logistic regression models simultaneously.

## Results

### Sample characteristics

Just over half of the adolescent sample was female (52%) and the mean age was 11.64 (SD 0.48) years. Table 1 presents the prevalence of not meeting individual health behaviour guidelines as well as the prevalence of combinations of behaviour according to adolescent gender. No statistical differences were found between boys and girls for individual or combined health behaviours. Seventy percent of participants exceeded the screen-time recommendations and 73.6% failed to consume sufficient fruits and vegetables. Almost 30% had none or one risk behaviour (3.9% of boys and 5.1% of girls had no risk behaviours, and 28.1% of boys and 22.6% of girls had one risk behaviour), and 27% had all three risk behaviours.

**Table 1:** Descriptive characteristics of adolescent participants (*n* = 527)

	All	Boys	Girls
N (%)	527	253 (48)	274 (52)
Age, years (mean (SD))	11.64 (0.48)	11.59 (0.49)	11.68 (0.47)
TV/DVD viewing (mins/day) (mean (SD))	190.98 (112.77)	192.55 (113.98)	189.56 (111.87)
> 120 min/day, %	69.7	70.2	70
Fruit and vegetable intake (frequency of consumption/day) (mean (SD))	3.77 (2.58)	3.80 (2.67)	3.75 (2.49)
< 5 a day (frequency of consumption/day), %	73.6	72.7	74.5
Energy-dense snack intake (frequency of consumption/day) (mean (SD))	3.77 (3.07)	3.93 (3.31)	3.62 (2.83)
> 2.8 a day (frequency of consumption/day), %	49.1	47.4	50.7
Risk behaviour groups, N (%)			
None or one risk behaviour	157 (29.8)	81 (32)	76 (27.7)
Low FV / high ED	43 (8.2)	21 (8.3)	22 (8)
High ST / low FV	134 (25.4)	62 (24.5)	72 (26.3)
High ST / high ED	50 (9.5)	23 (9.1)	27 (9.9)
3 risk behaviours (Low FV / high ST / high ED)	143 (27.1)	66 (26.1)	77 (28.1)

Pearson’s chi-square analyses between boys and girls for risk behaviour groups; Independent T-tests for comparison of means between boys and girls for all continuous variables

### Clustering of health behaviours

Eight possible combinations of the three health behaviours were examined, and the observed and expected prevalence ratios of these health behaviours and their combinations are displayed in Table 2. Overall, observed over expected ratios were close to 1 and ranged from 0.65 to 1.23. The three risk behaviour combination of insufficient fruit and vegetable consumption, high energy-dense snack consumption, and excessive screen-time occurred more frequently than expected (1.06 (1.01, 1.15)), as did the two risk behaviour combination of high energy-dense snack consumption and excessive screen-time, although not significant according to the confidence intervals (1.16 (0.98, 1.38)).

**Table 2:** Observed and expected prevalence of health risk behaviours, individually and in combination

No. of health behaviours	High TV/DVD	Low fruit and vegetable consumption	High energy-dense snack food consumption	O (%)	E (%)	O/E	(95% CI)
3	x	x	x	26.57	25.06	**1.06**	(1.01, 1.15)
2	x	x	-	25.81	25.93	0.99	(0.90, 1.08)
	-	x	x	7.02	10.71	0.65	(0.49, 0.82)
	x	-	x	10.44	8.98	**1.16**	(0.95, 1.38)
1	x	-	-	6.45	9.29	0.69	(0.51, 0.88)
	-	x	-	13.28	11.08	**1.20**	(1.02, 1.38)
	-	-	x	4.74	3.84	**1.23**	(0.84, 1.63)
0	-	-	-	4.55	3.97	**1.15**	(0.75, 1.54)

*O* observed prevalence; *E* expected prevalence; *95% CI* 95% confidence interval; *X* guideline not met; *−* guideline met

Bold: observed over expected ratios >1 are indicative of clustering

### Factors associated with combinations of adolescent risk behaviours

Additional file 1: Table S1, shows the description and distribution of the demographic, individual, behavioural, and social and physical home environmental variables. Several variables were associated with combinations of adolescent risk behaviours in the univariate multinomial logistic regression analysis (Table 3). The results of the multivariate multinomial logistic regression analysis are presented in Table 4. Results are described according to health behaviour combinations.

**Table 3:** Univariate multinomial logistic regression analysis of factors associated with combinations of risk behaviours among adolescents

	Low FV / high ED OR (95% CI)	High ST / Low FV OR (95% CI)	High ST / High ED OR (95% CI)	3 risk behaviours OR (95% CI)
Demographic				
Gender (ref: female)				
Male	0.89 (0.46–1.76)	0.81 (0.51–1.28)	0.80 (0.42–1.51)	0.80 (0.51–1.27)
Age (ref: age 12 years)				
11 years	0.61 (0.30–1.27)	0.84 (0.53–1.35)	0.87 (0.45–1.67)	0.65 (0.40–1.05)
Ethnicity (ref: Other)				
White / white British	2.08 (0.25–17.21)	1.07 (0.36–3.19)	0.80 (0.20–3.16)	0.53 (0.21–1.35)
Asian / Asian British	2.80 (0.30–26.56)	1.67 (0.50–5.59)	1.20 (0.30–5.61)	0.93 (0.32–2.70)
Brothers (ref: one or more)				
None	1.21 (0.61–2.43)	0.89 (0.54–1.45)	1.18 (0.61–2.28)	1.13 (0.70–1.80)
Sisters (ref: one or more)				
None	1.04 (0.53–2.04)	0.95 (0.60–1.52)	0.83 (0.43–1.60)	0.79 (0.50–1.26)
Parents at home (ref: other)				
Mother and Father	1.19 (0.55–2.66)	0.66 (0.40–1.08)	0.96 (0.48–1.92)	0.79 (0.49–1.30)
Deprivation scale (ref: low)				
Middle	1.44 (0.58–3.57)	0.92 (0.49–1.73)	1.93 (0.83–4.47)	1.21 (0.63–2.32)
High	0.97 (0.37–2.55)	1.07 (0.58–1.94)	1.44 (0.58–3.56)	1.86* (1.02–3.37)
Individual				
Habit for watching television (ref: low habit)	1.81 (0.88–3.73)	1.49 (0.93–2.42)	2.20 (1.12–4.29)*	2.29 (1.43–3.70)***
Habit for eating snack foods while watching TV (ref: low habit)	2.24 (1.09–4.62)*	1.23 (0.73–1.98)	3.62 (1.75–7.49)***	3.35 (2.04–5.51)***
Habit for eating FV while watching TV (ref: low habit)	0.95 (0.46–1.95)	1.12 (0.70–1.80)	2.84 (1.38–5.87)**	0.94 (0.59–1.49)
Self-efficacy for not watching TV/DVD’s or using computers (ref: low self-efficacy)	1.13 (0.55–2.29)	0.73 (0.45–1.18)	1.29 (0.66–2.50)	0.48 (0.30–0.77)**
Self-efficacy for not eating snack foods when watching TV/DVD’s (ref: low self-efficacy)	0.89 (0.44–1.80)	0.84 (0.52–1.36)	0.56 (0.28–1.11)	0.40 (0.24–0.66)***
Self-efficacy for increasing fruit and vegetable consumption (ref: low self-efficacy)	0.58 (0.28–1.20)	0.70 (0.42–1.15)	0.75 (0.38–1.48)	0.45 (0.27–0.73)***
Self-efficacy for reducing energy-dense snack food consumption (ref: low self-efficacy)	0.78 (0.39–1.55)	0.80 (0.50–1.30)	0.93 (0.48–1.80)	0.45 (0.28–0.72)***
Behavioural				
Eating breakfast while watching TV (ref: [2] or less days a week)	2.54 (1.24–5.20)**	1.35 (0.85–2.15)	3.26 (1.63–6.54)***	1.71 (1.08–2.70)*
Eating lunch while watching TV (ref: [2] or less days a week)	1.59 (0.80–3.16)	1.26 (0.79–2.01)	2.77 (1.40–5.50)**	1.89 (1.19–3.02)**
Eating dinner while watching TV (ref: [2] or less days a week)	1.58 (0.78–3.20)	1.75 (1.08–2.85)*	2.42 (1.19–4.93)**	2.24 (1.38–3.66)***
Eating fruit and vegetables while watching TV (ref: [2] or less days a week)	0.68 (0.34–1.39)	0.92 (0.57–1.47)	2.75 (1.37–5.52)**	0.86 (0.54–1.37)
Eating ED snacks while watching TV (ref: [2] or less days a week)	6.22 (2.92–13.27)***	2.14 (1.31–3.50)**	5.47 (2.70–11.09)***	11.45 (6.65–20.02***
Home social environment				
Eating dinner in front of the TV with parents (ref: less than twice a week)	1.67 (0.84–3.32)	1.60 (0.99–2.59)	2.66 (1.38–5.11)**	1.70 (1.06–2.71)*
Eating breakfast in front of the TV with parents (ref: less than twice a week)	1.56 (0.68–3.58)	1.40 (0.77–2.55)	3.16 (1.55–6.45)**	2.50 (1.44–4.35)***
Eating snacks in front of the TV with parents (ref: less than twice a week)	4.31 (2.09–8.87)***	1.69 (1.04–2.72)*	2.65 (1.38–5.08)**	3.99 (2.46–6.47)***
Watching TV/DVD’s together with parents (ref: less than twice a week)	2.05 (1.03–4.06)*	1.44 (0.89–2.30)	4.17 (2.07–8.38)***	1.72 (1.08–2.73)*
Parental food restriction (ref: low – below median score of 2.4)	0.53 (0.26–1.07)	0.52 (0.32–0.84)**	0.78 (0.40–1.54)	0.33 (0.20–0.56)***
Parental screen-time restriction (ref: low – below median score of 2)	0.46 (0.21–1.00)	0.80 (0.49–1.32)	0.76 (0.39–1.50)	0.43 (0.25–0.71)***
Home physical environment				
Television in the bedroom (ref: no)	1.88 (0.85–4.15)	1.77 (1.05–2.97)*	1.57 (0.77–3.19)	1.63 (1.01–2.68)*
Home availability of energy-dense snack foods (ref: low – below median score of 9)	5.83 (2.77–12.96)***	2.03 (1.18–3.50)**	4.07 (2.01–8.24)***	9.19 (5.31–15.90)***
Home availability of fruit and vegetables (ref: low – below median score of 6)	1.76 (0.84–3.68)	1.38 (0.97–2.77)	1.36 (0.70–2.67)	1.55 (0.96–2.51)
Home accessibility of energy-dense snack foods (ref: low – below median score of 8)	2.07 (0.97–4.40)	1.86 (1.13–3.06)*	1.96 (1.01–3.93)*	8.77 (4.53–16.95)***
Home accessibility of fruit and vegetables (ref: low – below median score of 4)	0.56 (0.27–1.17)	0.82 (0.50–1.34)	1.61 (0.79–3.27)	1.23 (0.75–2.01)

*p < 0.05; **p < 0.01; ***p < 0.001

**Table 4:** Multivariate multinomial logistic regression analysis of factors associated with combinations of risk behaviours among adolescents

	Low FV / high ED OR (95% CI)	High ST / Low FV OR (95% CI)	High ST / High ED OR (95% CI)	3 risk behaviours OR (95% CI)
Demographic				
Deprivation scale (ref: low)				
Middle	0.69 (0.19, 2.51)	0.60 (0.26, 1.39)	1.70 (0.41, 7.07)	0.83 (0.30, 2.24)
High	0.49 (0.12, 2.06)	0.73 (0.30, 1.77)	0.97 (0.23, 4.03)	1.23 (0.44, 3.41)
Individual				
Habit for watching television (ref: low habit)	1.37 (0.43, 4.36)	1.03 (0.47, 2.27)	1.58 (0.46, 5.36)	1.04 (0.43, 2.51)
Habit for eating snack foods while watching TV (ref: low habit)	1.75 (0.50, 6.14)	0.61 (0.28, 1.34)	2.72 (0.68, 10.89)	1.44 (0.56, 3.66)
Habit for eating FV while watching TV (ref: low habit)	1.58 (0.48, 5.19)	1.41 (0.68, 2.93)	3.05 (0.94, 9.94)	1.93 (0.79, 4.75)
Self-efficacy for not watching TV/DVD’s or using computers (ref: low self-efficacy)	1.55 (0.48, 5.00)	0.85 (0.40, 1.79)	2.28 (0.67, 7.77)	0.78 (0.31, 1.92)
Self-efficacy for not eating snack foods when watching TV/DVD’s (ref: low self-efficacy)	2.90 (0.78, 10.84)	1.08 (0.48, 2.42)	0.41 (0.11, 1.54)	1.39 (0.51, 3.76)
Self-efficacy for increasing fruit and vegetable consumption (ref: low self-efficacy)	0.87 (0.26, 2.90)	0.78 (0.37, 1.63)	0.44 (0.13, 1.48)	0.54 (0.22, 1.34)
Self-efficacy for reducing energy-dense snack food consumption (ref: low self-efficacy)	0.61 (0.17, 2.15)	1.51 (0.69, 3.33)	1.40 (0.41, 4.81)	0.79 (0.30, 2.06)
Behavioural				
Eating breakfast while watching TV (ref: [2] or less days a week)	6.12 (1.50, 25.35)**	0.96 (0.41, 2.25)	3.86 (0.96, 16.11)	1.91 (0.68, 5.35)
Eating lunch while watching TV (ref: [2] or less days a week)	0.33 (0.10, 1.41)	0.71 (0.26, 1.98)	0.27 (0.05, 1.42)	0.75 (0.24, 2.33)
Eating dinner while watching TV (ref: [2] or less days a week)	1.10 (0.24, 5.11)	1.76 (0.68, 4.59)	1.76 (0.31, 9.89)	1.34 (0.42, 4.34)
Eating fruit and vegetables while watching TV (ref: [2] or less days a week)	0.53 (0.16, 1.78)	1.05 (0.50, 2.19)	3.69 (1.04, 13.16)*	0.56 (0.23, 1.34)
Eating ED snacks while watching TV (ref: [2] or less days a week)	4.53 (1.24, 16.53)*	2.00 (0.89, 4.30)	1.37 (0.36, 5.33)	7.68 (2.75, 21.45)***
Home social environment				
Eating dinner in front of the TV with parents (ref: less than twice a week)	0.80 (0.21, 3.03)	1.24 (0.54, 2.89)	1.84 (0.48, 7.11)	0.88 (0.33, 2.41)
Eating breakfast in front of the TV with parents (ref: less than twice a week)	0.30 (0.06, 1.42)	0.90 (0.34, 2.40)	1.51 (0.36, 6.33)	0.66 (0.22, 1.99)
Eating snacks in front of the TV with parents (ref: less than twice a week)	6.64 (1.85, 23.85)**	1.24 (0.56, 2.74)	0.98 (0.29, 3.36)	1.86 (0.74, 4.67)
Watching TV/DVD’s together with parents (ref: less than twice a week)	0.63 (0.19, 2.03)	0.86 (0.41, 1.78)	4.50 (1.15, 17.57)*	1.17 (0.49, 2.79)
Parental food restriction (ref: low – below median score of 2.4)	0.70 (0.23, 2.15)	0.51 (0.25, 1.05)	2.42 (0.75, 7.78)	0.61 (0.26, 1.46)
Parental screen-time restriction (ref: low – below median score of 2)	1.17 (0.35, 3.93)	1.61 (0.77, 3.39)	1.46 (0.42, 5.05)	1.22 (0.50, 3.10)
Home physical environment				
Television in the bedroom (ref: no)	0.71 (0.21, 2.40)	1.78 (0.82, 3.86)	0.64 (0.17, 2.41)	0.87 (0.33, 2.27)
Home availability of energy-dense snack foods (ref: low – below median score of 9)	4.95 (1.45, 16.88)**	1.78 (0.82, 3.86)	3.22 (0.86, 12.16)	3.93 (1.52, 10.12)**
Home availability of fruit and vegetables (ref: low – below median score of 6)	1.83 (0.54, 6.22)	1.12 (0.48, 2.65)	0.87 (0.24, 3.09)	1.20 (0.48, 2.98)
Home accessibility of energy-dense snack foods (ref: low – below median score of 8)	0.86 (0.26, 2.82)	1.56 (0.75, 3.26)	5.64 (1.35, 23.59)**	4.24 (1.52, 11.80)**
Home accessibility of fruit and vegetables (ref: low – below median score of 4)				

*p < 0.05; **p < 0.01; ***p < 0.001

### All three risk behaviours

Thirteen variables were associated with an increased likelihood of reporting all three risk behaviours in the univariate analyses (Table 3), these included high deprivation, habit for watching TV, habit for eating snacks while watching TV, eating breakfast, lunch, dinner, and snacks while watching TV, watching TV and eating dinner, breakfast and snacks in front of the TV together with parents, having a TV in the bedroom and home availability and accessibility of energy-dense snack foods. Six variables were associated with a lower likelihood of reporting all three risk behaviours, these included self-efficacy to not watch TV, to not eat snacks while watching TV, to increase fruit and vegetable consumption, to decrease energy-dense snack food consumption, and parental food and screen-time restriction.

In the multivariate analyses, three variables remained significantly associated with an increased likelihood of reporting all three risk behaviours, these were eating energy-dense snacks while watching TV, and both the availability and accessibility of energy-dense snack foods within the home (Table 4).

### High ST/high ED

Fourteen variables were associated with an increased likelihood of reporting high ST / high ED snacks in the univariate analyses (Table 3). These included, habit for watching TV, habit for eating snacks while watching TV, habit for eating fruit and vegetables while watching TV, eating breakfast, lunch, dinner, fruit and vegetables, and snacks while watching TV, watching TV and eating dinner, breakfast, and snacks in front of the TV together with parents, and home availability and accessibility of energy-dense snack foods. In the multivariate analyses (Table 4), three variables remained significantly associated with an increased likelihood of reporting high ST/high ED, these were eating fruit and vegetables while watching TV, watching TV together with parents, and home accessibility of energy dense snack foods.

### High ST/low FV

Six variables were associated with an increased likelihood of reporting high ST / low FV in the univariate analyses (Table 3), these included, eating dinner and ED snacks while watching TV, eating snacks in front of the TV together with parents, having a TV in the bedroom, and home availability of energy-dense snack foods and fruit and vegetables. Parental food restriction was associated with a lower likelihood of reporting high ST/low FV.

No variables remained significantly associated with an increased likelihood of reporting high ST/low FV in the multivariate analyses.

### Low FV/high ED

Seven variables were associated with an increased likelihood of reporting low FV/high ED in the univariate analyses (Table 3), these included habit for eating snacks while watching TV, eating breakfast and ED snacks while watching TV, eating snacks in front of the TV together, watching TV together with parents, and home availability and accessibility of energy-dense snack foods.

Four of these variables remained significantly associated with an increased likelihood of reporting low FV/high ED in the multivariate analysis (Table 4), these were eating breakfast and ED snacks while watching TV, eating snacks in front of the TV together with parents, and home availability of ED snacks.

## Discussion

This study examined the prevalence, clustering, and correlates of screen-time and eating behaviours in a sample of young adolescents. Additionally, we investigated the individual, behavioural, and home social and physical environmental correlates of clustered health behaviours. Analyses revealed that behavioural risk factors are prevalent among young adolescents as the majority failed to meet guidelines for one or more health behaviour(s), Furthermore, high levels of screen viewing, low fruit and vegetable consumption, and high ED snack food consumption tend to cluster in this population. Our results show that individual, behavioural, and social and physical home environmental correlates were differentially associated with behavioural clusters. Correlates consistently associated with behavioural clusters included eating while watching TV, eating at the TV with parents, and the availability and accessibility of energy-dense snack foods within the home. Given the growing body of research which suggests that ‘unhealthy’ behaviours such as low fruit and vegetable consumption and sedentary screen-time co-occur as risk behavioural clusters in adolescents, and that the formation of clusters of unhealthy behaviours has the potential for synergistic negative effects on health outcomes, work such as ours, which aims to understand the clustering of health behaviours in young people, is important for preventative efforts.

In the current study at least 48% of adolescents failed to meet recommendations for each of the health behaviours studied and 27% of adolescents displayed all three risk behaviours. In contrast to previous studies no gender differences were found in either the prevalence of individual health behaviours or clusters of health behaviours [19]. Otherwise, our findings are similar to those of previous studies that have examined behavioural risk factors. For example, Elsenburg et al. [15] reported that around 30% of British adolescents failed to meet guidelines for five health behaviours including fruit and vegetable intake and screen-time, and Plotnikoff et al. [35] found that 43% of boys and 53% of girls from a sample of Canadian adolescents displayed two or more risk factors. In a US study which examined fruit and vegetable intake and screen-time in adolescents, Driskell et al. [36] reported that being at risk for one behaviour significantly increased the risk for another behaviour. Similarly, in a large European sample, increased television viewing and computer use was associated with lower fruit consumption [37]. While the trends across studies indicate a high prevalence of health risk behaviours among adolescents, direct comparison between studies is difficult due to the different methodologies undertaken to not only examine behaviours (which are diverse across studies), but also in the analysis to examine co-occurrence. Nonetheless, the evidence to date on the prevalence and clustering of these behaviours suggests that the further study of health behaviour risk patterns, as well as the investigation into understanding a wide range of correlates and determinants of these clusters of behaviours, is warranted to inform future prevention efforts.

The three risk behaviour combination of insufficient fruit and vegetable consumption, high energy-dense snack consumption, and excessive screen-time occurred more frequently than expected, as did the two risk behaviour combination of high energy-dense snack consumption and excessive screen-time. The clustering of high screen-time with unhealthy eating behaviours is consistent with previous findings from Hardy et al. who reported an observed/expected ratio of 2.3 (95% CI 1.3, 3.9) for high screen-time, low fruit and vegetable intake, and high soft-drink and snacking in adolescent girls [17], and with Elsenburg et al. who found an observed/expected ratio of 1.31 (95% CI 1.04, 1.59) for low physical activity, high screen-time, low fruit and vegetable intake and a high dietary fat/non-milk extrinsic sugar (MAR) score [15]. The odds of having multiple risk behaviours increase over the course of development, especially during the teenage years [11], and the risk of synergistic trajectories towards chronic disease [11–13] is likely greater than for individual health behaviours. It would be opportune, therefore, for efforts to be made to prevent the coupling of health behaviours at the time when they are likely to develop. Findings from our study indicate that such coupling is already highly prevalent by ages 11–12 years suggesting a need for preventative efforts prior to this age.

Identifying factors that could either increase the possibility of engaging in multiple risk behaviours or act as preventative factors is the first step towards targeted intervention efforts. To date, research examining factors associated with clustering of health behaviours has focussed on sociodemographic factors [19]. The present study builds upon the current evidence base by examining factors across multiple levels of the social-ecological model. High deprivation level was the only sociodemographic factor significantly associated with the three risk behaviour combination, and was only significant in the univariate model. In a recent review of the clustering of health behaviours in children and adolescents it was concluded that young people from a low socioeconomic status background were more likely to be in clusters defined by high levels of sedentary behaviour [19]. However, these conclusions were based on a small number of studies that were inconsistent not only in their assessments of dietary intake and sedentary behaviour, but in the factors used to define socioeconomic status. The lack of significance in the multivariate model in our study suggests that the inequalities identified in the univariate model are explained by the other significant factors in the multivariate model. This means that deprivation is likely related to these factors. Future research is warranted to understand the pathways to the significance of deprivation or socioeconomic status on clustered health behaviours in adolescents.

In addition to sociodemographic factors, the present study investigated individual, behavioural, and home social and physical environmental correlates of clustered health behaviours. No individual level factors were associated with clusters of health behaviours. Behavioural and home social and physical factors including energy-dense snacks seem to be key factors for adolescents reporting all three health risk behaviours and those reporting the two risk behaviour combination of low FV/high ED. Those with these combinations of two and three risk behaviours were more likely to be eating such ED snacks in front of the TV and have high availability and accessibility of these snacks in the home. Moreover, eating breakfast in front of the TV, and eating snacks with parents in front of the TV were also predictive of this two risk behaviour cluster membership. These findings advocate several broad intervention possibilities. First, it is prudent to reduce the availability and accessibility of energy-dense snacks in the home. Parents will have a clear role here and should negotiate with their children suitable and acceptable alternatives. Second, strategies are required to reduce food and meal consumption in front of the TV. The ideal meal option should be to have all TV and other electronic media switched off during mealtimes. Eating in front of the TV is likely to be a highly habitual behaviour developed over time through context-dependent repetition [38]. Strategies are required to break the habits of TV viewing and meal/snack consumption in front of the TV, by uncoupling the link between the behaviour and context. This might be done by planning TV programmes to watch rather than simply surfing for anything, and also by making snacks available only away from the context of the TV. We also need to understand why screen-time and unhealthy eating behaviours coexist in populations to address the root causes. Do some parents encourage these joint behaviours because they result in a happy quiet child when they are trying to do another task for instance? Do commercials on TV lead children to ask about snacks whilst watching TV or surfing the internet? There are a multitude of possible reasons for the clustering of these health behaviours. Qualitative research methods would allow us to better understand why these behaviours coexist to better design interventions that target changing the mediating variables associated with behaviours.

There are two clusters of dietary behaviours in the context of screen-time that have typically concerned health professionals. These concern screen-time and the consumption of energy-dense snacks and low fruit and vegetable consumption, as there is evidence linking screen-time and these unhealthy dietary behaviours [9, 10]. Results from the present study show that there was an increased likelihood of being in the high screen time and high ED cluster if adolescents watched TV with their parents and had high access to ED snacks in the home. Parental modelling of TV viewing has been associated with higher TV viewing in young people [39], hence one strategy to consider is that of targeting TV reduction in parents per se. To achieve this, suitable alternative behaviours must be found. Furthermore, home accessibility of healthy and unhealthy foods is a consistent predictor of healthy and healthy food consumption [40]. Limiting access to unhealthy snack foods in the home by either not having these types of snacks in the home or by providing increased accessibility to healthy snack options are strategies that warrant further investigation in the pursuit of reducing unhealthy snack food consumption and potentially uncoupling the cluster of screen-time and unhealthy eating behaviours.

Strengths of this study include the representative sampling methodology and the high response rate. Furthermore, a strength of this study is its inclusion of potential correlates from multiple levels of the social-ecological model, expanding the previous focus of literature in this field on sociodemographic factors. A limitation of this study is the use of self-reported questionnaires to assess behaviours. While this limitation is acknowledged, this method is common in this field, the administration of questionnaires was supervised by trained research staff, and validated questions were used where available. Future research studies in this area would be strengthened by the use of objective methods where possible. The cross-sectional nature of this study limits our ability to ascertain cause and effect. Longitudinal research examining the tracking of clusters of risk behaviours over time, as well as predictors of clusters, would strengthen this field of research that is currently dominated by cross-sectional studies.

## Conclusions

Recent reviews have confirmed the association between high screen time and less healthy dietary patterns in all age groups [9, 10], which is of public health concern as clusters of health behaviours are likely to have a greater impact on poor health than individual behaviours. However, it has not been easy to determine the correlates that might underline such patterns. The present study has addressed this gap by investigating correlates, at multiple levels of the social-ecological model, of combinations of behaviours, including screen time and diet. Findings of this study carry several important implications for public health practitioners. The study has shown that there is a high prevalence of screen-time and unhealthy eating in young adolescents, and that screen-time viewing is coupled with unhealthy dietary behaviours. Health promotion strategies and policies are required that address reductions in multiple health behaviours rather than the traditional focus on individual health behaviours. Efforts should be made to further examine and test modifiable factors associated with clusters of health behaviours, with a focus on alternative behaviours to replace some screen time, and changes to habitual dietary patterns, such as eating snacks and meals at the TV, and snack availability and accessibility in the home environment. These may require a combination of individual, social, and environmental changes that include parental behaviours, alongside conscious and more automatic (nudging) strategies.

## Additional file

Additional file 1: Table S1. Description and distribution (%) of demographic, individual, behavioural, and home social and physical environmental variables. (DOC 89 kb)

## Abbreviations

ED snacks: Energy-dense snacks
FV: Fruit and vegetables
SB: Sedentary behaviour
ST: Screen-time
TV: Television viewing
