# Supplementary material for: Clustering and correlates of screen-time and eating behaviours among young adolescents
Source: BMC Public Health. 2017 May 31;17:533. doi: 10.1186/s12889-017-4441-2 (PMC5452360; doi:10.1186/s12889-017-4441-2)
Supplement: Additional file 1: Table S1. — Description and distribution (%) of demographic, individual, behavioural, and home social and physical environmental variables. (DOC 89 kb) [file 12889_2017_4441_MOESM1_ESM.doc]

Table S1. Description and distribution (%) of demographic, individual, behavioural, and home social and physical environmental variables

|  | **Total (n=527)** |
| --- | --- |
| **Demographic** | |
| ***Ethnicity*** |  |
| White / White British | 77.1 |
| Asian / Asian British | 17.1 |
| Other | 5.8 |
| ***Siblings*** |  |
| One or more brother | 64.2 |
| No brothers | 35.8 |
| One or more sister | 58.9 |
| No sisters | 41.1 |
| ***Parents at home*** |  |
| Live with mother and father | 67.4 |
| Other | 32.6 |
| ***Deprivation scale*** |  |
| Low (least deprived) | 40.8 |
| Medium | 26.1 |
| High (most deprived) | 33.2 |
| **Individual** | |
| ***Habits*** | |
| Habit for watching television (median score: 2.75) |  |
| Low | 45.6 |
| High | 54.4 |
| Habit for eating snack foods while watching TV (median score: 3) |  |
| Low | 41.3 |
| High | 58.7 |
| Habit for eating fruit and vegetables while watching TV (median score: 3) |  |
| Low | 44.8 |
| High | 55.2 |
| ***Self-efficacy*** |  |
| Self-efficacy for not watching TV/DVD’s or using computers (median score: 2.83) |  |
| Low | 52.6 |
| High | 47.4 |
| Self-efficacy for not eating snack foods when watching TV/DVD’s (median score: 3) |  |
| Low | 60.5 |
| High | 39.5 |
| Self-efficacy for increasing fruit and vegetable consumption (median score: 3) |  |
| Low | 59.8 |
| High | 40.2 |
| Self-efficacy for reducing energy-dense snack food consumption (median score: 2.67) |  |
| Low | 51.3 |
| High | 48.7 |
| **Behavioural** | |
| Eating breakfast while watching TV^1^ |  |
| Low | 45.9 |
| High | 54.1 |
| Eating lunch while watching TV^1^ |  |
| Low | 45.3 |
| High | 54.7 |
| Eating dinner while watching TV^1^ |  |
| Low | 35.4 |
| High | 64.6 |
| Eating fruit and vegetables while watching TV^1^ |  |
| Low | 51.7 |
| High | 48.3 |
| Eating energy-dense snacks while watching TV^1^ |  |
| Low | 44.9 |
| High | 55.1 |
| **Social environment** | |
| Eating dinner in front of the TV with parents^2^ |  |
| Low | 56.2 |
| High | 43.8 |
| Eating breakfast in front of the TV with parents^2^ |  |
| Low | 75.3 |
| High | 24.7 |
| Eating snacks in front of the TV with parents^2^ |  |
| Low | 50.2 |
| High | 49.8 |
| Watching TV/DVD’s together with parents^2^ |  |
| Low | 51.3 |
| High | 48.7 |
| Parental food restriction (median score: 2.4) |  |
| Low | 59.2 |
| High | 40.8 |
| Parental screen-time restriction (median score: 2) |  |
| Low | 68 |
| High | 32 |
| **Physical environment** | |
| Television in bedroom |  |
| Yes | 68.9 |
| No | 31.1 |
| Home availability of energy-dense snack foods (median score: 9) |  |
| Low | 55.2 |
| High | 44.8 |
| Home availability of fruit and vegetables (median score: 6) |  |
| Low | 62.1 |
| High | 37.9 |
| Home accessibility of energy-dense snack foods (median score: 3) |  |
| Low | 30.5 |
| High | 69.5 |
| Home accessibility of fruit and vegetables (median score: 8) |  |
| Low | 39.8 |
| High | 60.2 |

^1^Low = ‘2 or less days a week’; High = ‘3 or more days a week’

^2^Low = less than twice a week’; High = ‘2 or more times a week’
